# Left-Leggedness
*Read before the members of the British Association at Newcastle-on-Tyne.


**Published:** 1889-09-21

**Authors:** W. K. Sibley


### "LEFT-LEGGEDNESS."*

By Dr. W. K. Sibley.

Professor Ball, in "Le Dualisme Cérébral," speaks of man as a right-handed animal. Being right-handed, it is popularly assumed that he is also right-legged; but this does not appear to be the case. Standing working with the right hand there is a tendency to use the left leg for balance. Many people find less exertion in going round circles to the right than to the left. Race-paths are nearly always made for running in circles to the right. So the majority of movements are more readily performed to the right, as dancing, running, etc. The rule in walking is to keep to the right, and this appears to be almost universal. It is more natural to bear to the right. Of a large number of people from the better-educated classes asked about the existence of the rule only 67 per cent. males and 53 per cent. females were aware of the rule; the large majority obey it unconsciously in walking. Crowds tend to bear to the right. The left leg being the stronger, it is more readily brought into action; hence troops start off with the left foot; it is the foot which is placed into the stirrup of the saddle or step of bicycle in mounting; so the left is the foot which a man takes off from in jumping. The experiments of Mr. G. H. Darwin blindfolding boys and telling them to walk straight, the right-handed one diverged to the right, and *vice versâ*. From measurements of Dr. Garson of the skeletons of the two legs, 54·3 per cent., the left was the longer and 35·8 the right. For measurements of the feet the author collected the drawings and measurements of 200 pairs, for which he is indebted to the courtesy of Mr. Parker, of Oxford-street, with the result that in 44 per cent. the left was longer, in 21·5 per cent. the right, and in 34·5 per cent. they were the same size. Measurement at the first joint gave 56 per cent. left larger, and at the instep 42·5 per cent. From the table of the figures it is observed that the left foot is more frequently the larger in the male than female sex, and the percentage of feet of the same size is greater in the female. The percentage of the right larger than the left is very constant, whereas the numbers of the left larger and those in which both feet were the same size are much more variable. Man, being naturally or artificially right-handed and left-legged, tends unconsciously to bear to the right; lower animals, on the other hand, appear nearly always to circle to the left.

* Read before the members of the British Association at Newcastle-on-Tyne.

Table Showing Difference in the Size of the Feet.

| | | Length. | First joint. | Instep. | Heel. |
|---|---|---|---|---|---|
| Left larger ... | A | 48 | 59 | 43 | 44·5 |
| | B | 38 | 50 | 40 | 16 |
| | C | 44 | 56 | 42·5 | 33 |
| Right larger... | A | 22 | 24 | 28·5 | 34 |
| | B | 20 | 24 | 22 | 34 |
| | C | 21·5 | 24 | 26·5 | 36·5 |
| Same size ...... | A | 30 | 17 | 26·5 | 21·5 |
| | B | 42 | 26 | 38 | 50 |
| | C | 34·5 | 20 | 30 | 30·5 |

(A) Percentage of 150 males, (B) percentage of 50 females, (C) percentage of 200 mixed cases.

